# Young HIV-Infected Children and Their Adult Caregivers Prefer Tablets to Syrup Antiretroviral Medications in Africa

**DOI:** 10.1371/journal.pone.0036186

**Published:** 2012-05-02

**Authors:** Patricia Nahirya-Ntege, Adrian Cook, Tichaona Vhembo, Wilfred Opilo, Rachel Namuddu, Richard Katuramu, Jessica Tezikyabbiri, Bethany Naidoo-James, Diana Gibb, on behalf of the ARROW Trial Team

**Affiliations:** 1 MRC/UVRI Uganda Research Unit on AIDS, Entebbe, Uganda; 2 MRC Clinical Trials Unit, London, United Kingdom; 3 University of Zimbabwe Medical School, Harare, Zimbabwe; 4 Joint Clinical Research Centre, Kampala, Uganda; 5 Baylor-Uganda Paediatric Infectious Disease Clinic, Mulago Hospital, Kampala, Uganda; University of Ottawa, Canada

## Abstract

**Background:**

Provision of anti-retroviral therapy (ART) for HIV-infected children is complicated using syrup formulations, which are costlier than tablets, harder to transport and store and difficult for health-workers to prescribe and caregivers to administer. Dispersible/crushable tablets may be more appropriate. We studied the acceptability of syrups and scored tablets among young children who used both in the AntiRetroviral Research fOr Watoto (ARROW) trial.

**Methods:**

ARROW is an ongoing randomized trial of paediatric ART monitoring and treatment strategies in 1206 children in Uganda and Zimbabwe. 405 children initially received syrups of combination ART including Nevirapine, Zidovudine, Abacavir and Lamivudine before changing, when reaching the 12-<15 kg weightband, to scored adult-dose tablets prescribed according to WHO weightband tables. Caregiver expectations and experiences were collected in questionnaires at their last visit on syrups and after 8 and 24 weeks on tablets.

**Results:**

Questionnaires were completed by caregivers of 267 children (median age 2.9 years (IQR 2.5, 3.4)). At last visit on syrups, 79% caregivers reported problems with syrups, mostly related to number, weight, transportation and conspicuousness of bottles. Difficulties taking tablets were expected by 127(48%) caregivers; however, after 8 and 24 weeks, only 26% and 18% reported their children had problems with tablets and no problems were reported with transportation/conspicuousness. Taste, swallowing or vomiting were reported as problems ‘sometimes/often’ for 14%, 9%, 22% children on syrups and 16%, 9%, 8% on tablets. At last visit on syrups, 74% caregivers expected to prefer tablets but only 27% thought their child would. After 8/24 weeks, 94%/97% caregivers preferred tablets and 57%/59% reported their child did.

**Conclusions:**

Most children at about 3 years can take tablets; caregivers and children themselves generally prefer tablets to liquid formulations of HIV medications above this age. Preferences of caregivers and children should be considered when designing and licensing paediatric drug formulations.

## Introduction

The paediatric management of long term anti-retroviral therapy (ART) involves interactions between children, their caregivers, other family members and healthcare providers around administration of antiretroviral drugs. For adults, good adherence to medication is central to the success of ART [1], [2], [3]; for young children, who rely on caregivers to administer drugs, the process is further complicated by lower availability of simple paediatric formulations for children of different ages [4].

In resource-limited settings, oral solution formulations present particular challenges [5]; they are up to four times more costly than solid formulations [6], [7], [8], and so for many illnesses, children may be given reduced doses of adult formulations. Oral solutions are also bulky, making them harder to transport and store, and are more conspicuous than tablets which may result in problems with confidentiality for patients receiving drugs used to treat HIV infection [5].

Solutions and syrups are administered using syringes, and different volumes for individual drugs are required. This, in addition to the need to increase volume repeatedly as the child grows, may present considerable challenges for caregivers, particularly for elderly carers who often have problems seeing markings on syringes in the absence of glasses. Further, whereas fixed dose formulations of anti-retroviral (ARV) drugs are available as combination tablets [9], [10], [11], fixed dose combination solutions are generally not available, so each has to be given separately. Accurate measurement of adherence to solutions or syrups through drug return at pharmacy level is also very challenging, requiring weighing of bottles [12].

Major problems with the palatability of some antiretroviral oral solutions have also been reported, in some cases requiring administration through gastrostomy tubes [13], [14], [15]. To improve palatability solutions may contain high levels of sugar; however this may worsen dental health which has already been reported as a problem in HIV-infected children [16].

Therefore, tablets which can be dispersed, crushed or chewed may be preferable to solution or syrup formulations, even for very young children, provided the correct dose can be given. Procurement of child and adult formulations may be harmonised, with fewer formulations required, if partial tablets can be given; this requires tablets with active drug uniformly distributed and scored for easy and accurate division. WHO have produced weightband tables of preferred doses (calculated from body surface area) of single and fixed dose combination solid formulation ARV drugs of available doses to simplify the process of prescribing paediatric ART by healthcare workers (Tables 1 and 2) [17].

**Table 1:** Dosing of NRTI antiretroviral drugs in the ARROW trial using WHO weightbands based on body surface area [17].

Weight (kg)	Abacavir				Lamivudine				Zidovudine			
	syrup		tablets		syrup		tablets		syrup		tablets	
	20 mg/ml		300 mg		10 mg/ml		150 mg		10 mg/ml		300 mg	
	a.m.	p.m	a.m.	p.m	a.m.	p.m	a.m.	p.m	a.m.	p.m	a.m.	p.m
5 to ≤7	3 ml	3 ml			3 ml	3 ml			7 ml	7 ml		
7 to ≤10	4 ml	4 ml			4 ml	4 ml			9 ml	9 ml		
10 to ≤12	5 ml	5 ml			5 ml	5 ml			10 ml	10 ml		
12 to ≤15	6 ml	6 ml	½	½	6 ml	6 ml	½	½	12 ml	12 ml	½	½
15 to ≤17			½	½			½	½			½	½
17 to ≤20			½	½			½	½			½	½
20 to ≤25			1	½			1	½			1	½
25 to ≤30			1	1			1	1			1	½
>30			1	1			1	1			1	1

**Table 2:** Dosing of NNRTI antiretroviral drugs in the ARROW trial using WHO weightbands based on body surface area [17].

Weight (kg)	Nevirapine*				Efavirenz		
	syrup		tablets		capsule	capsule	tablet
	10 mg/ml		200 mg		50 mg	200 mg	600 mg
	a.m.	p.m.	a.m	p.m.	daily	daily	daily
5 to ≤7	7 ml	7 ml					
7 to ≤10	9 ml	9 ml					
10 to ≤12	10 ml	10 ml				1	
12 to ≤15	11 ml	11 ml	½	½		1	
15 to ≤20			1	½	1	1	
20 to ≤25			1	½			½
25 to ≤30			1	1	1		½
30 to ≤40			1	1		2	
>40			1	1			1

• Dose escalation at half dose shown for first 14 days.

Despite the numerous practical problems associated with syrups, licensing authorities frequently promote the need for liquid formulations during childhood, and there is a general lack of data on the formulation preferences of caregivers and/or children. In this study we evaluated the acceptability of syrup and divided scored solid tablet formulations of antiretroviral drugs in children weighing more than 12 kg who changed from syrups to tablet ARV formulations. This is a substudy of the ARROW trial (www.arrowtrial.org; ISCRTN 24791884), an ongoing randomised trial of monitoring practice and first-line induction-maintenance ART strategies in ART-naïve HIV-1 infected children in Uganda and Zimbabwe, in which pharmacokinetic studies have demonstrated that scored adult-dose tablets, dosed according to WHO weightband tables, provide correct plasma ARV drug levels for children over 3 years [18], [19], [20].

## Methods

The study was conducted in four centres in Uganda and Zimbabwe: the Joint Clinical Research Centre, Kampala; Baylor College of Medicine Children's Foundation at Mulago Hospital, Kampala, and the Medical Research Council, Entebbe; and at the University of Zimbabwe Clinical Research Centre, Harare. The protocol was approved by the ethics committee local to each study centre and by the University College London ethics committee in the UK. All caregivers gave written informed consent.

The parent ARROW trial is an open-label randomized factorial trial comparing two monitoring strategies and three treatment strategies. Children were randomized to either clinically driven monitoring (CD4 tests never returned to treating clinicians; toxicity tests undertaken but only returned to clinicians if clinically indicated), or laboratory plus clinical monitoring (3-monthly CD4 tests and haematology/biochemistry monitoring blood tests, all returned to clinicians). For treatment, children were randomized to either a continuous two nucleoside reverse transcriptase inhibitor (NRTI) plus one non-nucleoside reverse transcriptase inhibitor (NNRTI) regimen, or one of two induction:maintenance regimens in which they took an additional NRTI for the first 36 weeks.

Antiretroviral drugs were supplied by ViiV GlaxoSmithKline and national programmes as individual paediatric syrups and as scored adult tablets (PK shown to be appropriate when administered dispersed in water or crushed [19]) for children >12 kg according to WHO weightband dosing tables.

Between March 2007 and November 2008, 1206 HIV-1 infected ART-naïve children aged 3 months to 17 years were enrolled in the ARROW trial. Of these, 405 children weighed less than 12 kg and initiated ART with syrups because parts of individual adult tablet doses were not available for lower weightbands [17]. On reaching 12 kg, study doctors would suggest to caregivers that their child could now take halved scored tablets instead of syrups with no change to dose or dosing schedule. When caregivers were happy to change, the doctor would then prescribe tablets. The ways in which tablets could be given, such as being dispersed in liquids, were explained to caregivers by pharmacists; children were not specifically coached on how to swallow tablets. The majority of children (64%, n = 171) took tablets dissolved or crushed and administered with a small amount of liquid. After starting tablets, care-givers were also free to change their children back to syrups at any time.

From June 2008, acceptability questionnaires were administered by trial nurses at the time of changing from syrups to tablets, and 8 and 24 weeks later. Nurses administering questionnaires were not involved in the decision to change to tablets; this was made by the caregiver with a study doctor. The initial questionnaire elicited the caregivers' experience with syrups and their expectations of tablets; follow-up questionnaires asked about their actual experience with tablets. Structured questions were used to record difficulties with syrups, problems (such as taste, swallowing, vomiting) anticipated with tablets, problems experienced with tablets, and the type of formulation preferred by caregivers and their children (parents/caregivers reported their view of the childrens' preferences). The same questions about problems were asked for both syrups and tablets. The questionnaires were completed in English by nurses who asked the questions in the local language when necessary; questionnaires were not translated because English is widely used by both adults and children in the urban areas of Uganda and Zimbabwe.

Statistical significance of differences between groups was evaluated with t-tests for continuous variables and Chi-square tests for categorical variables. Stata software, version 11.1 (StataCorp), was used in all analyses.

## Results

405 children initiated ART using syrups. Of these, 63 changed to tablets before June 2008 when the acceptability substudy began; 5 remained on syrups at the time of analysis in August 2011 (all less than or recently attained 12 kg), and 27 had left the study before changing formulation (20 died, 7 were lost to follow-up). Of the 310 eligible children who changed from syrups to tablets between June 2008 and August 2011, 21 did not have questionnaires administered in error and 22 completed only one questionnaire. This analysis therefore includes 267 (267/310 = 86%) children who changed from syrups to tablets with data available at the time of switch and 8 weeks later. At the time of analysis, 91% of children changing to tablets (244/267) had been on them for more than 24 weeks and had completed the 24-week questionnaires.

The median age of children when they changed from syrups to tablets was 2.9 years (IQR 2.5, 3.4), having started syrups at median age 1.7 years (1.1, 2.3) and spent median 1.1 (0.7, 1.6) years on ART (Table 3). Children taking tablets whole, rather than crushed or dispersed, were slightly older (mean age 3.3 v. 2.9 years, p<0.001). Of note, no families declined to change from syrups to tablets and the oldest child was 6 years at formulation change. There were similar numbers of boys and girls (55% girls, n = 146). At ART initiation, severe wasting and stunting were common (weight-for-age Z-score median −3.1, IQR −4.4,−2.1; height-for-age Z-score median −3.1, IQR −4.1,−2.2) but on ART considerable recovery had occurred before changing to tablets (Table 3). Similarly, most children were moderate to severely immuno-compromised at the time of ART initiation (pre-ART CD4% median 13.6% (IQR 10%,19%); CD4 count median 794 (516,1184)), but substantial increases had occurred on ART before changing to tablets (at change to tablets CD4% median 32% (26%, 38%); CD4 count median 1409 cells/mm3 (975, 2078)). Most children had quite advanced HIV disease at ART initiation; 50% (n = 134) and 25% (n = 66) had experienced WHO stage 3 and stage 4 events respectively.

**Table 3:** Participant characteristics, 267 children.

	At start of syrups		At change to tablets	
	Median	IQR	Median	IQR
Age (years)	1.7	(1.1, 2.3)	2.9	(2.5, 3.4)
Sex, n (%) male	121	(45%)		
				
Weight-for-age Z score	−3.1	(−4.4, −2.1)	−1.2	(−1.8, −0.5)
Height-for-age Z score	−3.1	(−4.1, −2.2)	−2.7	(−3.2, −1.7)
				
CD4%	13.6%	(10%, 19%)	32%	(26%, 38%)
				
WHO stage 3, n(%)	134	(50%)		
WHO stage 4, n(%)	66	(25%)		
				
Person bringing child, n(%)				
Mother	197	(74%)		
Father	16	(6%)		
Grandmother	28	(10%)		
Aunt	17	(6%)		
Other	9	(4%)		

### Caregiver expectation of tablets

Before changing formulation, approximately half the caregivers expected to encounter difficulties administering tablets to their children (48%, n = 127), in particular because of taste (30%, n = 80), swallowing (25%, n = 66) or vomiting (19%, n = 51) which they expected might be difficult with tablets. Problems were anticipated slightly more frequently by those who reported problems while giving syrups, but the difference between groups was not statistically significant (50%, n = 105 of those reporting problems with syrups expected problems with tablets v. 40%, n = 22 among others, p = 0.21).

### Experience with syrups

At the time of changing from syrup to tablet formulations, 212 (79%) primary caregivers reported they had experienced problems either sometimes or often while using syrups. Most difficulties related to managing the large number of bottles supplied by the pharmacy (reported by 47%, n = 123), the weight of bottles (63%, n = 166), and transporting bottles (58%, n = 152) (Table 4). A child on three-drug ART weighing 10–12 kg would typically require seven bottles of syrup at each 28-day visit and therefore considerably more with less frequent visits. Anecdotally, clinic staff had noticed caregivers bringing increasingly large bags to clinics in order to carry syrups home.

**Table 4:** Problems experienced sometimes or often: using syrups; first 8 weeks of tablets; first 24 weeks of tablets.

	Syrups,	n = 267		Tablets,	n = 267	(8 wks)	Tablets,	n = 244	(24 wks)
	n	(%)	N	n	(%)	N	n	(%)	N
Timing of dose	23	(8.6)	267	4	(1.5)	263	5	(2.0)	244
Measuring syrup/Counting tablets	24	(9.0)	267	2	(0.7)	265	2	(0.8)	244
Taste	38	(14.3)	266	42	(15.7)	225	31	(12.7)	244
Swallowing	25	(9.4)	265	24	(9.0)	243	11	(4.5)	243
Vomiting	58	(22.0)	264	22	(8.4)	239	17	(7.0)	242
Taking the whole dose	10	(3.8)	264	5	(1.9)	256	8	(3.3)	242
Storage	21	(8.0)	263	0	(0.0)	261	1	(0.4)	242
Number of bottles	123	(46.8)	263	0	(0.0)	261	2	(0.8)	242
Weight of bottles	166	(63.1)	263	1	(0.4)	260	2	(0.8)	242
Transporting bottles	152	(57.6)	264	0	(0.0)	261	2	(0.8)	242
Losing/breaking bottles	24	(9.1)	264	0	(0.0)	261	1	(0.4)	242
Identifying different drug bottles	11	(4.2)	264	4	(1.5)	257	2	(0.8)	242
Keeping syrup cool	3	(1.1)	262		N/A			N/A	
Conspicuousness	33	(12.5)	264	3	(1.1)	258	3	(1.2)	242
Cleaning the syringes	26	(9.8)	264		N/A			N/A	
Losing the syringes	8	(3.0)	264		N/A			N/A	
Opening tablet bottles		N/A		3	(1.1)	258	1	(0.4)	242

### Experience with tablets

**Figure 1:**
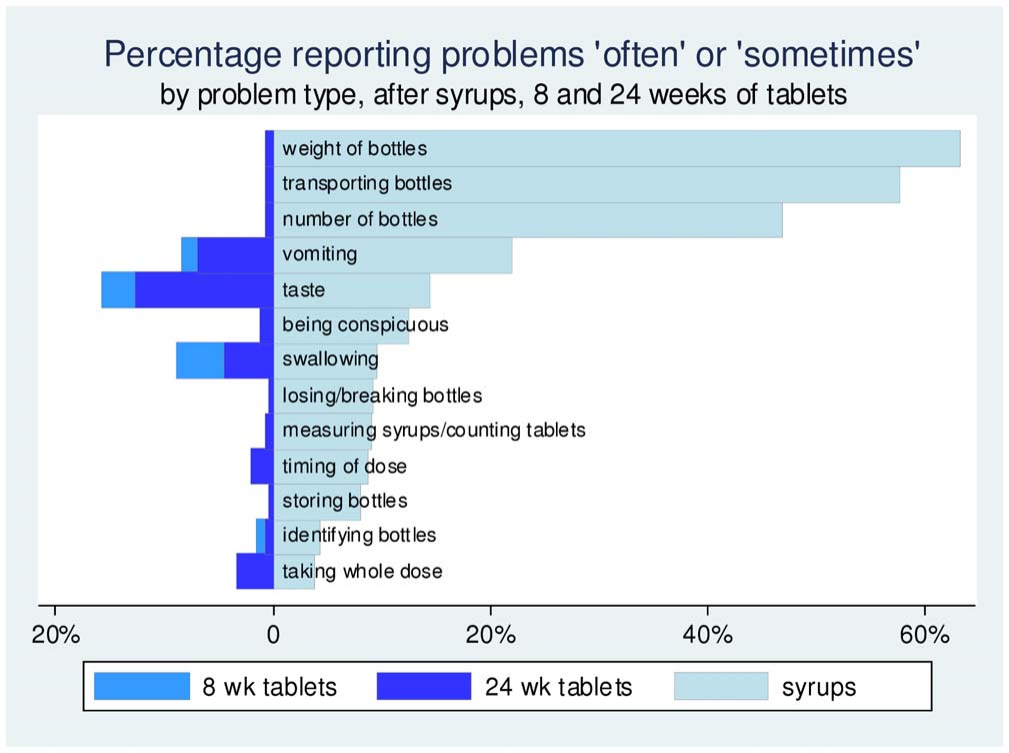
Problems ‘sometimes’ or ‘often’ with syrups and tablets.

After 8 weeks of administering tablets, 69 (26%) caregivers reported problems, declining to 45 (18%) after 24 weeks. The most frequently reported difficulties decreased between 8 and 24 weeks: from 16% to 13% for tablet taste, 9% to 5% for swallowing difficulties, and 8% to 7% for vomiting. At 8 weeks, only problems with taste were reported more frequently with tablets than with syrups and this reversed by week 24 (Figure 1). Problems during the first 8 weeks of tablets were no more likely to occur among children previously having problems with syrups (Table 5); however 21% (n = 41) of those with problems continuing at 24 weeks had experienced problems with syrup, compared to 8% (n = 4) among those not having problems with syrups (p = 0.04).

**Table 5:** Problems after 8 and 24 weeks on tablets, by problems with syrups.

	8 weeks of tablets					24 weeks of tablets			
Syrup	problem	no problem	N	p	Syrup	problem	no problem	N	p
**problem**	51 (26%)	144 (74%)	195		problem	41 (21%)	154 (79%)	195	
**no problem**	13 (27%)	36 (73%)	49	0.96	no problem	4 (8%)	45 (92%)	49	0.04
**N**	64	180	244		N	45	199	244	

244 children with 8 and 24 week questionnaires.

### Overall preference

**Figure 2:**
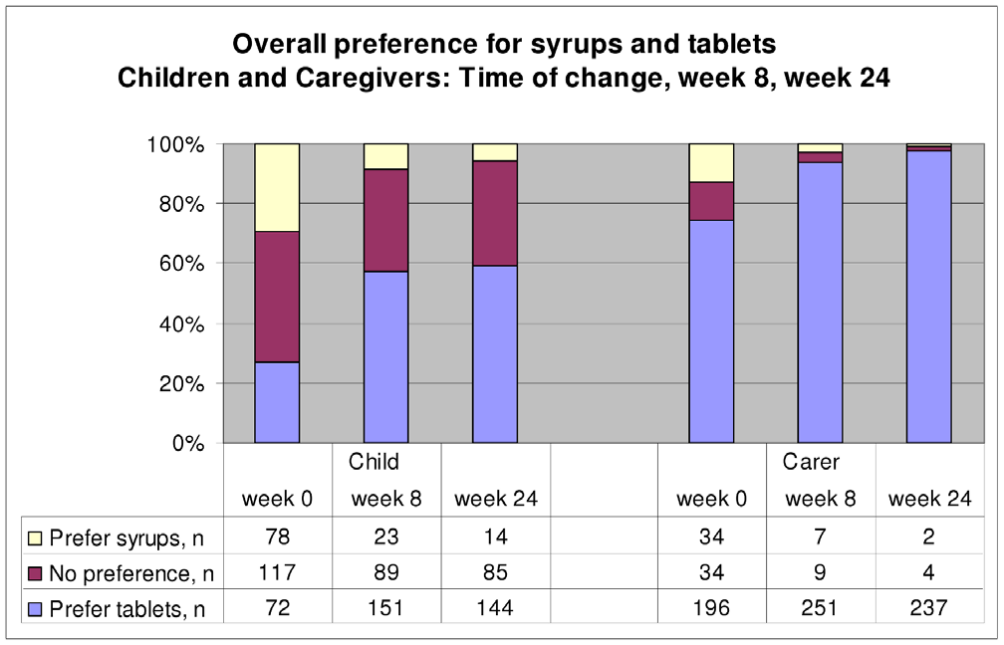
Overall preference for syrups and tablets.

Before using tablets, caregiver opinion was divided about which formulation they thought their child would prefer (27% tablets, 29% syrups, 44% no preference (Figure 2). However, after 8 weeks on tablets, more than half the caregivers reported a preference among their children for tablets (57% tablets, 9% syrups, 34% no preference), with a similar preference at 24 weeks (59% tablets, 6% syrups, 35% no preference). The age of the 23 children who preferred syrups after 8 weeks using tablets was similar to the 151 children reported to prefer tablets (mean age 2.9 v. 3.1 years, p = 0.20); however, all those preferring syrups were less than 4 years of age. Of note, no child changed from tablets back to liquids during the 24 weeks follow-up.

When asked about preference from their own (as opposed to their child's) perspective, caregivers questioned before formulation change were clear that they thought they would prefer tablets (74% tablets, 13% syrups, 13% no preference). After 8 weeks on tablets, actual preference increased for tablets (94% tablets, 3% syrups, 3% no preference), and after 24 weeks nearly all caregivers preferred tablets (97% tablets, 1% syrups, 2% no preference).

## Discussion

There are few studies comparing the acceptability of tablet and syrup formulations of drugs for caregivers and their children; for antiretroviral drugs, this is particularly important because treatment is life-long and adherence is critical for successful outcome. In our study of young children in a resource-limited setting, we report a preference for tablets, which increased with time from starting tablets. Since children were young at the time of changing from syrups to tablets (around 3 years of age), we asked caregivers about the preference of their children, and also their own preference as caregivers. We found that most children, at about 3 years, can take tablets and that caregivers and children themselves generally prefer tablets to liquid formulations of HIV medications above this age.

In a small qualitative study, also in the field of HIV infection, Bagenda et al. [21] reported that caregivers preferred giving their children tablets rather than syrups. Reasons for disliking syrups were problems with measurement, spillage and the bulkiness of single drug formulations, very similar to the practical problems reported by caregivers in our study. In a larger quantitative study the same authors reported significantly better adherence using tablets instead of syrups [22], based on pharmacy refill data. A study by Yeung et al. [23] found that HIV-infected children changed formulation at around 7 years in the UK; one would expect the acceptability of tablets to increase with age during childhood but the narrow ageband (IQR 2 to 3 years) made this difficult to assess in our study. Preferences and acceptability were not formally or prospectively evaluated in the UK study and it can only be assumed, as children were switched to ‘off label’ adult formulations (which would not then have been licensed for children and may not have been scored), that this was undertaken in consultation with caregivers and it may have been driven by children or carers experiencing problems taking syrups.

Pill swallowing skills can be learned by children at relatively young ages. Garvie et al reported that US children as young as 4 years could be taught to take tablets, contrary to initial caregiver concerns and expectations [24], while Thomson et al reported increasing ability to swallow minitablets with age in children aged 2 to 5 years [25]. The narrow ageband of children in this study prevented analysis of swallowing technique and age; however most caregivers crushed or dispersed tablets in a small amount of liquid. Of note, the package insert did not formally state that drugs were dispersible, but this was demonstrated by study investigators before the study commenced, and pharmacokinetic data were satisfactory when administering drugs this way to the same children [19]. Taste, swallowing and vomiting were the key difficulties expected by the caregivers of children changing to tablets; however, these difficulties appeared no worse for tablets than they had been for syrups and by 24 weeks on tablets few problems were reported, and these were mainly in children who had also had problems with syrups. For 14 children the change from syrups to tablets coincided with the planned reduction from 4 to 3 drugs in their induction:maintenance regimen; while their preference for tablets may have been inflated by this change, any impact on overall results is likely to be small. The strength of the preference for tablets is perhaps best informed by the fact that no child in this study changed from tablets back to syrups, although this option was available. Syrup formulations used in this study did not require refrigeration; it seems likely that this would have further strengthened the preference for tablets, a further consideration with increasing use of first-line protease inhibitors where syrups need refrigeration.

Reasons for caregivers preferring tablets appeared to relate mainly to practical issues such as transportation and conspicuousness in the household, as recognition of antiretroviral drugs could be associated with HIV infection. In general, the proportion of caregivers who preferred tablets was consistently higher than the proportion who said their children preferred them. As caregivers were reporting for their children, it is possible that caregivers' preference for tablets could have influenced their reporting of specific child-related problems in favour of tablets.

Young HIV-infected children starting ART depend on adults for their care and adherence to medication. Bagenda et al reported that the biggest challenge felt by caregivers was the commitment to administering lifelong ART [21]. The difficulties faced by caregivers while using liquid formulations should therefore be considered alongside the perceived preferences of the children themselves. If caregivers' experience of giving ART can be improved by moving children to tablets at an early age, this could improve adherence and thus efficacy. However, we are unaware of any trials comparing outcomes of randomising children to continue liquid formulations or to move to solid-based formulations. In ARROW we are collecting adherence data on both syrups and tablets, although direct comparison may be limited by differences in data collection methods. This highlights another problem with the use of liquid formulations in resource-limited settings as the weighing of bottles is time-consuming, requires complex calculations and is likely to be subject to greater error than pill counts. Further, prescribing exact amounts of syrup required between each visit is more difficult than using tablets.

The simplification and standardisation of ART is an essential feature of HIV treatment scale up and rollout to lower level health centres in rural areas where large numbers of people with HIV live. In fact, the WHO promotes Flexible Solid Oral Dosage forms (FSOD) as part of it's ‘Make Medicines Child Size’ initiative; these are dosage forms that can be swallowed whole or split, or can be dispersed in liquid prior to administration. Wherever possible, formulations of new HIV drugs with potential for use in children should be scored, (although, of note, WHO recommends no more than a single scoreline for HIV drugs to produce 2 equal halves as “quarters” scoring is likely to be impractical and result in errors). The WHO weightband dosing tables were designed to be used across all anti-retroviral drugs, with the dose of each drug fixed (e.g. ½ tablet am/pm) for any given weightband, thus reducing the complexity of prescribing for healthworkers, as well as administration by caregivers as all drug doses change at the same time. Weightband doses for the NRTI drugs were calculated using the licensed recommendations for whole or half adult tablets made by ViiV Glaxosmithkline, validated by pharmacokinetic studies [18], [19], [20], and which have been licensed by EMA in Europe and FDA in US. Dosing of tablets using such weightband tables provides a simple alternative compared to dosing of syrups which are calculated using mg per kg for some drugs (eg abacavir or lamivudine) and mg per m^2^ body surface area for others (zidovudine), which is more likely to be associated with dosing errors [26]. Affordable fixed-dose combination (FDC) solid scored tablets of several antiretroviral drugs are also now available and licensed for children, and provide an even easier way for caregivers to administer and health workers to prescribe ARVs [17], [27], [28].

Liquid formulations may be needed for treatment of infants, although some ‘baby’ FDCs can be given to infants as small as 3 kg. Our large study provides evidence to challenge those advocating for syrup formulations beyond age 3 years. Organisations such as the Drugs for Neglected Diseases initiative (DNDi), Medecins sans Frontieres (MSF) and the United Nations Childrens Fund (UNICEF) are all working on the development of closer links between pharmaceutical companies, regulators, paediatricians and families to promote more timely development of appropriate paediatric formulations and better harmonisation between formulation needs of children and adults in order to simplify supply chain logistics for implementers of ARV programmes. Thus, drug manufacturers (both innovator and generic) should be encouraged to formally address issues which might impact on successful scoring early in development. These include the content uniformity (to allow pills to be split), pharmacokinetic characteristics (Cmax, Cmin and AUC) when tablets are taken whole or when split (i.e. supportive formal PK studies to demonstrate bioequivalence), and dispersiblity in water.

In summary, scored tablets of ARVs for children as young as three years cause few problems and most children and almost all caregivers prefer them. The high acceptability of the tablets by caregivers may help adherence thereby improving efficacy and durability of treatment among young children who face life-long ART. In addition, tablets can reduce logistic problems for health system providers, simplify prescribing for healthcare workers and reduce the burden of care for caregivers. We would argue that scoring adult tablets so they can be easily and accurately broken should be considered at an early stage in drug development if it is anticipated that children will also benefit; this might also reduce the time taken for drugs to be licensed for use in children. Preferences of caregivers and their children should be considered when designing and licensing paediatric drug formulations, particularly for resource-limited settings where most HIV-infected children live.

## References

[1] Bangsberg DR, Perry S, Charlebois ED, Clark RA, Roberston M (2001). Non-adherence to highly active antiretroviral therapy predicts progression to AIDS.. AIDS.

[2] Gross R, Yip B, Lo Re V, Wood E, Alexander CS (2006). A simple, dynamic measure of antiretroviral therapy adherence predicts failure to maintain HIV-1 suppression.. J Infect Dis.

[3] Nachega JB, Hislop M, Dowdy DW, Lo M, Omer SB (2006). Adherence to highly active antiretroviral therapy assessed by pharmacy claims predicts survival in HIV-infected South African adults.. J Acquir Immune Defic Syndr.

[4] Sohn A, Ananworanich J (2007). How can we simplify antiretroviral therapy in children?. Curr Opin HIV AIDS.

[5] Havens PL, Gibb DM (2007). Increasing antiretroviral drug access for children with HIV infection.. Pediatrics.

[6] UNICEF, WHO (2010). Sources and prices of selected medicines for children.. http://www.who.int/medicines/publications/essentialmedicines/Sources_Prices2010.pdf.

[7] WHO Global Price Reporting Mechanism Transaction prices for antiretroviral medicines and HIV diagnostics from 2008 to October 2009.. http://www.who.int/hiv/amds/GPRMsummaryReportNov2009.pdf.

[8] UNICEF, WHO (2004). Improving access to appropriate paediatric ARV formulations.. http://www.who.int/3by5/en/finalreport.pdf.

[9] Calmy A, Pascual F, Ford N (2004). First-line and second-line antiretroviral therapy.. The Lancet.

[10] Gilks CF, Crowley S, Ekpini R, Gove S, Perriens J (2006). The WHO public-health approach to antiretroviral treatment against HIV in resource-limited settings.. The Lancet.

[11] Pujari SN, Patel AK, Naik E, Patel KK, Dravid A (2004). Effectiveness of Generic Fixed-Dose Combinations of Highly Active Antiretroviral Therapy for Treatment of HIV Infection in India.. J Acquir Immune Defic Syndr.

[12] Walker A, Ford D, Mulenga V, Thomason M, Nunn A (2009). Adherence to Both Cotrimoxazole and Placebo is Associated with Improved Survival Among HIV-Infected Zambian Children.. AIDS Behav.

[13] Shingadia D, Viani RM, Yogev R, Binns H, Dankner WM (2000). Gastrostomy tube insertion for improvement of adherence to highly active antiretroviral therapy in pediatric patients with human immunodeficiency virus.. Pediatrics.

[14] Temple ME, Koranyi KI, Nahata MC (2001). Gastrostomy tube placement in nonadherent HIV-infected children.. Ann Pharmacother.

[15] King JR, Yogev R, Aldrovandi G, Chadwick E, Acosta EP (2004). Pharmacokinetics of antiretrovirals administered to HIV-infected children via gastrostomy tube.. HIV Clin Trials.

[16] Eldridge K, Gallagher J (2000). Dental caries prevalence and dental health behaviour in HIV infected children.. Int J of Paediatr Dent.

[17] WHO (2010). Antiretroviral therapy for HIV infection in infants and children: towards universal access. Recommendations for a public health approach (2010 revision).

[18] Fillekes Q, Natukunda E, Balungi J, Kendall L, Bwakura-Dangarembizi M (2011). Paediatric under-dosing of Efavirenz: a pharmacokinetic study in Uganda.. J Acquir Immune Defic Syndr.

[19] Kasirye P, Kendall L, Adkison KK, Tumusiime C, Ssenyonga M (2012). Pharmacokinetics of oral solution versus tablet formulation varies with antiretroviral drug in the target population of HIV-1 infected children.. Clinical Pharmacology and Therapeutics.

[20] Musiime V, Kendall L, Bakeera-Kitaka S, Snowden W, Odongo F (2010). Pharmacokinetics and acceptability of once versus twice daily lamivudine and abacavir in HIV-1 infected Ugandan children in the ARROW trial.. Antivir Ther.

[21] Bagenda A, Kallander K, Barlow-Mosha L, Byogero R, Musoke P (2010). Caregiver experience with syrup and tablet formulations of HAART for paediatric HIV treatment in an urban clinic [Abstract CDB0228]..

[22] Bagenda A, Barlow-Mosha L, Bagenda D, Sakwa R, Fowler MG (2011). Adherence to tablet and liquid formulations of antiretroviral medication for paediatric HIV treatment at an urban clinic in Uganda.. Ann Trop Paediatr.

[23] Yeung VW, Wong ICK (2005). When do children convert from liquid antiretroviral to solid formulations?. Pharm World Sci.

[24] Garvie PA, Lensing S, Rai SN (2007). Efficacy of a Pill-Swallowing Training Intervention to Improve Antiretroviral Medication Adherence in Pediatric Patients With HIV/AIDS.. Pediatrics.

[25] Thomson SA, Tuleu C, Wong I, Keady S, Pitt K (2009). Minitablets: New modality to deliver medicines to preschool-aged children.. Pediatrics.

[26] Livshits Z, Lee S, Hoffman R, Nelson L, Esteban-Cruciani N (2011). Zidovudine overdose in a healthy newborn receiving postnatal prophylaxis.. Clin Toxicol.

[27] Mulenga V, Cook A, Walker AS, Kabamba D, Chijoka C (2010). Strategies for Nevirapine Initiation in HIV-Infected Children Taking Pediatric Fixed-Dose Combination “Baby Pills” in Zambia: A Randomized Controlled Trial.. Clin Infect Dis.

[28] Chokephaibulkit K, Plipat N, Cressey T, Frederix K, Phongsamart W (2005). Pharmacokinetics of nevirapine in HIV-infected children receiving an adult fixed-dose combination of stavudine, lamivudine and nevirapine.. AIDS.

